# Editorial: When children also shape the family environment

**DOI:** 10.1002/jcv2.70136

**Published:** 2026-05-29

**Authors:** Eivind Ystrom

**Affiliations:** ^1^ Promenta Research Center Department of Psychology University of Oslo Oslo Norway; ^2^ Department for Child Health and Development Norwegian Institute of Public Health Oslo Norway; ^3^ Centre for Research on Equality in Education Faculty of Educational Sciences University of Oslo Oslo Norway

**Keywords:** adolescence, family environment, mental health, network analysis

## Abstract

Family research in child and adolescent mental health has often been framed as if influence runs mainly from adults to children. Bjørndal et al. provide a timely corrective. Using longitudinal network models that separate within‐person from between‐person associations, they show that adolescent mental health difficulties and family dynamics are interrelated over time, and that emotional symptoms may precede later hyperactivity/inattention and sibling problems. Their findings also point to reinforcing loops within the family system, particularly between low family support and negative feelings towards the family. This matters because family members are not merely environmental exposures; they are genetically correlated social environments, and children can shape parental and sibling dynamics as well as be shaped by them. Distinguishing between variables with stronger outgoing and ingoing influence may help refine which intervention hypotheses should be prioritized, and which should be discarded or treated more cautiously.

For decades, developmental theory has emphasized that families are transactional systems rather than one‐way pipelines of influence (Kretschmer, 2023). Yet much empirical work in child and adolescent mental health still reads as though parents act and children respond. That framing is understandable, but incomplete. Children and adolescents do not simply receive family environments; they also shape them. Bjørndal et al. (2026) bring that point into sharp focus by examining how adolescent mental health difficulties and family environment factors move together over time within individuals, rather than only across families that differ from one another in more stable ways.

This is an important step. As Bjørndal et al. (2026) note, many studies of family factors and youth mental health have been cross‐sectional, have focused on one narrow exposure at a time, or have conflated between‐family and within‐family associations. That matters because the question most relevant for developmental science and intervention is often not whether adolescents from more supportive families differ from adolescents from less supportive families (Speyer et al., 2022). The more difficult and more useful question is whether, when a given adolescent experiences a shift in symptoms or family dynamics, other parts of the family system shift thereafter as well.

Bjørndal et al. (2026) show that the answer is yes, but not in a simple or uniform way. Emotional symptoms predicted later increases in hyperactivity/inattention and more problems with siblings. Lack of family support and negative feelings towards the family formed a reciprocal loop over time. Conduct problems clustered more with externalized family dynamics, such as fighting with parents and bothering siblings, whereas emotional symptoms were tied more closely to internalized family experiences, such as feeling negative about one's family and being bothered by siblings. This is not merely another reminder that “family matters.” It is evidence that different symptom domains may connect to different family processes, and that siblings deserve a more central place in family models of adolescent mental health (Valstad et al., 2026).

The broader significance of this study becomes clearer when placed alongside genetically informed family research. Parents provide both their children's rearing environments and half of their segregating DNA, meaning that parent‐child associations are often entangled with gene‐environment correlation (Cheesman et al., 2023). Genetically informative studies have therefore emphasized that parent‐offspring associations cannot be interpreted straightforwardly as parental environmental effects unless shared genetic liability and direction of effects are taken seriously (Jami et al., 2021). Just as importantly, child characteristics can evoke responses from caregivers. In other words, child‐to‐parent effects are not a nuisance detail. They are part of the phenomenon.

Bjørndal et al. (2024) have previously used genotyped families to showcase that family members' heritable traits may matter through the environment. Bjørndal et al. (2026) sheds light and color on their previous findings: Family members are not just background context; they are genetically correlated environments. The same family system can therefore contain parent‐to‐child effects, child‐to‐parent effects, sibling processes, shared liabilities, and feedback loops, all at once.

Seen in that light, one of the most promising features of the new paper is its distinction between variables with stronger outgoing and ingoing influence in the temporal network. Bjørndal et al. (2026) found that emotional symptoms, fighting with parents, and lacking family support showed stronger outward influence, whereas negative feelings towards the family and hyperactivity/inattention were more strongly predicted by other variables in the model. This distinction is useful because it can help sort intervention hypotheses. A variable with stronger outgoing influence may be a better candidate leverage point. A variable with stronger ingoing influence may be better understood as a downstream indicator, or as an outcome that is especially sensitive to change elsewhere in the system.

Importantly, this does not mean that outgoing estimates identify intervention targets in any definitive sense. Bjørndal et al. (2026) are appropriately cautious, and so should we be. Their model used roughly two‐year intervals, and the authors note that different patterns might emerge at shorter timescales. That caution is well aligned with broader transactional research showing that reciprocal links between parental support and adolescent depressive symptoms may differ across daily, bi‐weekly, annual, and biennial intervals (Boele et al., 2023). Outgoing and ingoing estimates are therefore best treated as tools for creating and discarding hypotheses, not as substitutes for causal inference. They can tell us where to look next, but not yet what will work in practice.

That distinction has practical value. It can help the field avoid overinvesting in attractive but weak ideas, while giving better theoretical priority to hypotheses that deserve triangulation in genetically informed, quasi‐experimental, or intervention designs. In that respect, the present paper fits well with Bjørndal and colleagues' broader programme of work suggesting that stressful life events within and between families display distinct prediction of internalising disorders (Bjorndal et al., 2023). The lesson across these studies is not that one design solves the problem. It is that stronger developmental inferences come from convergence across methods.

Bjørndal et al. (2026) therefore make two contributions at once. Empirically, they show that emotional symptoms, family support, parent conflict, and sibling problems belong in the same developmental conversation. Conceptually, they remind us that the family environment should not be treated as an inert backdrop. Families are dynamic systems of reciprocal influence, and those systems are partly genetically patterned. If the field takes that seriously, we will move beyond asking whether families matter, and toward asking how influence flows through families, for whom, and on what timescale. That is exactly the kind of question that can improve both developmental theory and the next generation of intervention hypotheses.

## AUTHOR CONTRIBUTIONS

**Eivind Ystrom**: Writing—original draft; writing—review and editing.

## CONFLICT OF INTEREST STATEMENT

E.Y. is a joint editor of *JCPP Advances*. The author declares no conflicts of interest.

## ETHICAL CONSIDERATIONS

Ethical approval was not required for this Editorial article.

## Data Availability

Data sharing not applicable to this article as no datasets were generated or analysed during the current study.
